# Fatigue Life Mapping of Rubber Isolators Based on Maximum Strain Energy Density and Cyclic Energy Dissipation Criteria with Specimen Data

**DOI:** 10.3390/polym18141732

**Published:** 2026-07-15

**Authors:** Yupeng Du, Jinying Huang, Zhenfang Fan, Jiaolin Wei, Wenwen Zhang, Xiaolong Wang

**Affiliations:** 1 College of Electromechanical Engineering, North University of China, Taiyuan 030051, China; bg20240112@st.nuc.edu.cn; 2 College of Mechanical Engineering, North University of China, Taiyuan 030051, China; fanzf@nuc.edu.cn (Z.F.); jlw3219@163.com (J.W.); xiaolongzhiwang@163.com (X.W.); 3 College of Computer Science and Technology, North University of China, Taiyuan 030051, China; zww931017@163.com

**Keywords:** natural rubber, life, fatigue damage parameter, strain energy density, cyclic energy dissipation

## Abstract

The ride stability and driving comfort of vehicles are highly dependent on the performance of the damping system. The fatigue life prediction of damping components using rubber as the core damping material has become a research hotspot in the field of vehicle vibration isolation. Taking an automotive engine rubber isolator as the research carrier, this paper jointly carries out finite element simulation analysis and structural component fatigue life tests. A dual-parameter mapping framework is proposed, which integrates maximum strain energy density and cyclic energy dissipation instead of using a single damage indicator. This approach comprehensively accounts for the coupling effect of energy storage and energy dissipation coexisting under actual service conditions. Through uniaxial tensile tests on rubber specimens, combined with finite element simulations and physical model parameters, a quantitative mapping relationship between laboratory specimens and full-scale engine rubber isolators is established. Based on this mapping, the fatigue life curve of the isolator is derived from the specimen-based failure characteristics. Validation tests under two randomly selected operating conditions yield prediction errors of 7.5% and 6.9%, demonstrating that the proposed model can accurately achieve equivalent fatigue life transformation from small specimens to actual components. Unlike conventional direct extrapolation methods, this approach does not require complex multiaxial fatigue tests on the component itself; it only needs simple specimen fatigue data, significantly reducing development costs, while providing a reliable theoretical basis for material selection, fatigue performance optimization, and forward structural design of rubber isolators.

## 1. Introduction

Automotive engine rubber mounts are predominantly deployed in service scenarios subjected to reciprocating cyclic loads demanding ultra-long fatigue life, which renders the investigation into mechanical fatigue loading of rubber vibration dampers a research focus of paramount importance [1,2,3]. In the design and development phase, experimental and theoretical research on rubber specimens and raw rubber materials constitutes a pivotal approach to effectively enhancing the fatigue performance of finished products.

For the entire engine mount system, rubber materials serve as the core functional medium, undertaking the fundamental role of absorbing and dissipating cyclic vibrational and impact energy transmitted from the engine. This energy mitigation capability not only bolsters the operational stability and driving safety of vehicles [4] but also effectively isolates the feedback dynamic effects from the vehicle chassis on the engine assembly [5]. Endowed with long molecular chains and a dense cross-linked network structure, rubber materials exhibit a distinct hysteretic energy-absorbing effect under reciprocating cyclic loading, which originates from the relative slip and internal friction of molecular chains [6,7,8]. This intrinsic material characteristic enables the conversion of harmful mechanical energy into thermal energy via hysteresis loss; while absorbing and attenuating high-frequency vibrational energy, it also suppresses the resonance phenomenon induced by engine excitation [2], thereby realizing the dual functions of vibration and noise reduction.

Extensive research efforts have been devoted to the fatigue life prediction of rubber mounts both domestically and internationally, and a relatively complete theoretical and experimental system has been established for studies centered on natural rubber as the primary vibration isolation material [9,10,11,12]. Thermo-oxidative aging is one of the main causes of rubber material failure. To address this phenomenon, Li et al. [13] studied the aging behavior of rubber materials under both high and low temperature conditions, while Li et al. [14] found through comparison of different rubber materials that the aging of natural rubber is the mildest under high temperature environments. Building on the research achievements of predecessors, the Miner cumulative damage model [15,16] has become a mainstream research paradigm for characterizing the fatigue damage evolution of rubber materials, where the cumulative damage variable is defined based on the total energy absorbed or dissipated by the material during cyclic loading. This model is predominantly established on the basis of uniaxial fatigue tensile tests, a methodological approach that offers significant advantages in saving time and economic costs for the fatigue life design and evaluation of rubber products [17]. Belkhiria et al. [18] established fatigue life prediction models for rubber materials based on logarithmic strain, engineering strain, Green–Lagrange strain, Euler strain, and octahedral shear strain, and validated the accuracy of multiple models through uniaxial tensile fatigue tests on styrene-butadiene rubber (SBR). Zhu et al. [19] showed that using the maximum strain energy density as a fatigue life evaluation parameter for rubber isolators yields relatively accurate results under uniaxial fatigue test conditions. However, when components are subjected to more complex loading conditions, Ayoub et al. [20] compared the capabilities of several different damage parameters for predicting the multiaxial fatigue life of rubber. Through combined multiaxial tension–compression and torsion tests, they found that the maximum principal strain could not adequately reflect the fatigue life of rubber, and pointed out that strain energy density is not a valid fatigue parameter for predicting the fatigue life of SBR. Robert [21] conducted a comprehensive comparison of six types of damage criteria under a full range of loading conditions and found that the two criteria based on the effective tensile strain $\varepsilon_{t}$ and effective shear strain $\gamma_{t}$, defined by the three principal strain components and the three shear strain components, respectively, yielded relatively good results. However, a sound damage criterion should not only predict the degree of damage but also the crack orientation.

With further development in materials research, energy dissipation, calculated by numerically integrating the experimental stress–strain results using the measured axial load and torque, has been adopted as a damage parameter for predicting fatigue crack nucleation [22,23]. Zine et al. [24] used energy dissipation as a damage parameter for fatigue life assessment under uniaxial tension and pure shear fluctuating loads, and found that strain energy density (SED) appears to be a reliable damage indicator. Banić et al. [25] investigated the main modes of energy dissipation in rubber under cyclic loading. In early studies, Ro [26] evaluated the comparative effectiveness of different damage parameters for uniaxial fatigue life prediction. Wang et al. [27] studied the energy dissipation characteristics of fiber-polyethylene composites under cyclic loading. Lin et al. [28] proposed that, since internal friction of molecular chains contributes to energy loss, energy dissipation can be enhanced by using polymers with an improved transition region. Zhou et al. [29] carried out bending tests on glass fiber–resin composites using ABAQUS and analyzed energy loss based on the energy dissipation angle. Wang et al. [30] investigated the absorbed energy density and energy dissipation of GF/VE composites through Brazilian splitting tests.

Ding et al. [31] constructed a rubber fatigue life prediction model based on cumulative fatigue damage theory, using the tearing energy range as the damage parameter. Dong et al. [32] predicted the fatigue crack initiation life of rubber isolators based on the material’s S-N curve. Mika et al. [33] proposed a parameter identification method based on the Gaussian mixture model (GMM) to obtain the constitutive relations and dissipative energy density functions of direction-dependent materials. Gosar et al. [34], based on continuum damage mechanics and incorporating mean stress correction, proposed a novel multiaxial energy approach that combines elastic strain variation with complementary energy transformation to introduce a new energy-based damage indicator. Gan et al. [35] conducted data-driven fatigue life research on metallic materials based on mapping relationships. Tobajas et al. [36] proposed a new fatigue life prediction method that simultaneously considers stress, strain, and strain energy.

However, when only the maximum strain energy density is used as the damage parameter for actual rubber isolators, the prediction errors under cyclic tests are significant, making it necessary to introduce a life prediction model. Cyclic energy dissipation is also a key parameter reflecting the performance of rubber isolators. At present, most evaluation parameters only consider a single physical quantity, i.e., either strain energy density or cyclic energy dissipation, and thus fail to fully characterize the fatigue damage mechanism of rubber materials under actual service conditions where energy storage and energy dissipation coexist. It should be noted that even combining these two parameters does not constitute an ideal damage criterion, as they cannot effectively predict the degree of damage or the direction of crack propagation. However, since the objective of this study is the failure time of rubber specimens, and the period from the initiation of damage to the onset of crack growth accounts for only a small fraction of the total life, leveraging the advantages of both parameters to establish a mapping relationship for vulnerable locations can effectively overcome this limitation. To this end, through basic uniaxial tensile tests of rubber materials, a mapping relationship of “maximum strain energy density + cyclic energy dissipation” is established. Combined with finite element simulation results and physical model parameters, a quantitative mapping relationship between laboratory specimens and actual engineering products is constructed. Based on this relationship, the fatigue life curve of critical components is fitted. Two operating conditions are randomly selected for testing, with test data recorded and fitting results calculated simultaneously; corresponding verification tests are then conducted to evaluate the model and validate the research framework. This study aims to provide a more comprehensive and accurate theoretical basis for fatigue life prediction and the structural optimization design of rubber engine mounts in vehicles.

## 2. Materials and Methods

**Figure 1:**
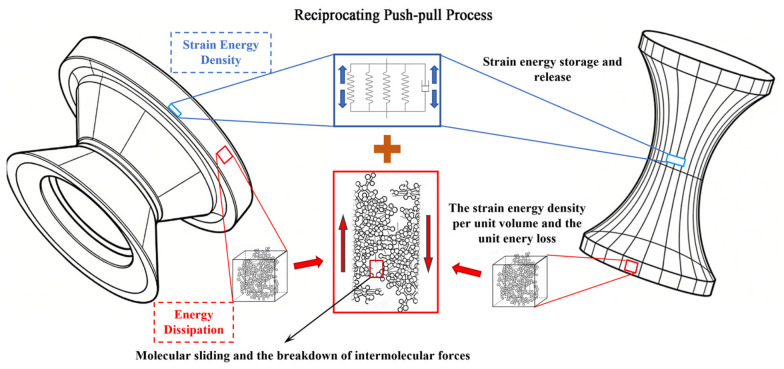
Energy transformation diagram for rubber specimens under uniaxial conditions.

This study focuses on rubber test columns and rubber isolators as the core research objects, with the structural characteristics shown in Figure 1 serving as the primary analytical basis. Under reciprocating tensile and compressive loading, the two types of rubber configurations exhibit distinct mechanical behaviors, including strain energy storage, release, and energy dissipation. Among these, energy dissipation is the key factor inducing rubber structural failure, whereas strain energy storage and release have a relatively minor impact on fatigue life. Nevertheless, the amount and spatial distribution of stored strain energy can accurately reflect the weak and damaged regions of the rubber structure, and can also provide timely responses when over-range loading conditions occur. Based on these mechanical principles, this paper integrates the characteristics of strain energy storage/release with the energy dissipation mechanism to systematically characterize the intrinsic evolutionary behavior of rubber structures during reciprocating tension–compression motion, thereby establishing a mapping relationship between structural response and fatigue life.

### 2.1. Strain Energy Density

In the reciprocating motion of structures, the dominant forms of energy in rubber materials are elastic energy storage and energy dissipation induced by the hysteresis phenomenon. In previous research, strain energy density has been adopted as the primary damage parameter for rubber materials. This is attributed to the fact that the damage caused by the intrinsic energy dissipation of rubber materials during reciprocating motion is typically negligible and does not constitute the primary factor leading to rubber damage. Additionally, other scholars have analyzed the temperature rise in rubber resulting from this energy dissipation as a cause of rubber material aging: temperature is the dominant factor accelerating rubber aging, thereby increasing the probability of rubber material damage.

Rubber is a hyperelastic nonlinear material, and its strain energy density can be calculated using the formula dW=σ:dε. In this equation, the subscripts σ and ε denote a pair of conjugate parameters, corresponding to the stress tensor and the strain tensor, respectively, which together fully characterize the stress and strain states at a given material point. Under the uniaxial loading conditions adopted in this experiment, the formula can be further reduced to the expression [37] $W=\int_{0}^{\varepsilon_{ij}}\sigma_{ij}d\varepsilon_{ij}$, where the parameter $\sigma_{ij}$ represents stress.

As can be inferred from the above equation, the calculation of energy density requires the operation of tensors. However, tensor calculations are typically overly abstract and cumbersome. Therefore, this article introduces the equivalent elastic strain to equate the stress tensor at the vulnerable locations, which can also reduce the computational complexity.

Owing to the focus of this study being on the damaged regions of rubber vibration dampers, a preliminary assumption is made for the experiment that the locations of maximum stress and strain in the rubber material correspond to its most vulnerable regions. On this basis, the aforementioned theoretical derivation and calculation process can be further simplified. Following the same equivalence logic as above, the equivalent elastic strain can be derived based on the von Mises yield criterion as follows:

(1) $$W_{d}=\frac{1}{2}\Delta \sigma_{\max}\cdot \Delta \varepsilon_{\max}$$

The calculation in this section aims to identify the vulnerable regions of the rubber material. Since rubber is a homogeneous material, the positions where stress and strain are the highest correspond to the most damage-prone locations. Therefore, the von Mises criterion is adopted in this paper to simplify and derive the stress and strain tensors, and the specific form is given as follows:

(2) $$W_{d}=\sqrt{\frac{1}{8}{\left[{\left(\sigma_{1}-\sigma_{2}\right)}^{2}+{\left(\sigma_{2}-\sigma_{3}\right)}^{2}+{\left(\sigma_{3}-\sigma_{1}\right)}^{2}\right]}_{\max}\cdot {\left[{\left(\varepsilon_{1}-\varepsilon_{2}\right)}^{2}+{\left(\varepsilon_{2}-\varepsilon_{3}\right)}^{2}+{\left(\varepsilon_{3}-\varepsilon_{1}\right)}^{2}\right]}_{\max}}$$

where $\sigma_{1},\sigma_{2},\sigma_{3}$ denote the three principal strains, corresponding to the diagonal values of the stress tensor; $\varepsilon_{1},\varepsilon_{2},\varepsilon_{3}$ denote the three principal strains, corresponding to the diagonal values of the strain tensor. Thus, it can be concluded that if the maximum equivalent strain and stress results are consistent, the maximum strain energy density at the most vulnerable locations of the two components (rubber test columns and rubber vibration dampers) under this working condition will also be consistent. Combined with the Mooney–Rivlin (MR) model established in Section 2 of this article, by applying the corresponding loads to the rubber test columns and rubber vibration dampers, the following key data at the vulnerable locations of the rubber material can be obtained.

### 2.2. Energy Dissipation

The energy dissipation of rubber is primarily attributed to its viscoelasticity: under the action of alternating external loads, a portion of the mechanical energy is converted into thermal energy and dissipated [38]. Scholars in the global mechanical and polymer fields have generally simplified this viscoelastic behavior into a coupled model of a viscous dashpot and a Hookean spring. As shown in the lower part of Figure 1, the dissipative component mainly originates from the relative slippage and friction of molecular chains, which are induced by the phase difference between stress and displacement in the rubber material.

When rubber materials are subjected to harmonic alternating stress, the formula for energy dissipation per unit volume (specific energy consumption) within a single loading-unloading cycle is expressed as [39]:

(3) $$\Delta W_{t}=\pi \cdot \sigma_{0}\cdot \varepsilon_{0}\cdot \sin\delta$$

where $\Delta W_{t}$ is the energy dissipation per unit volume generated in one loading cycle; $\sigma_{0}$ is the maximum value of cyclic stress; $\varepsilon_{0}$ is the alternating strain amplitude; and sinδ is the loss angle, which refers to the phase difference between stress and strain caused by hysteresis.

**Figure 2:**
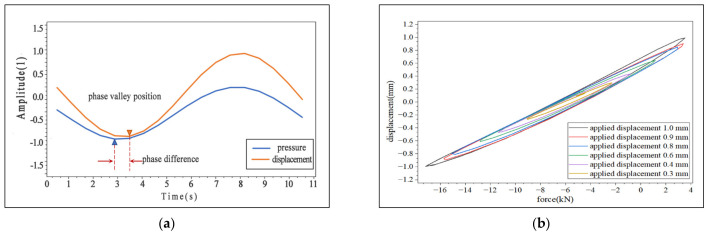
Stress–strain hysteresis phase difference for rubber vibration damper (**a**), and hysteresis curves of rubber vibration damper (**b**).

Cyclic mechanical tests were conducted on the vibration-damping component and rubber test columns, respectively. As illustrated in Figure 2a, a phase difference exists between stress and displacement, which is the key factor leading to energy dissipation. In the figure, the *x*-axis represents the entire tension–compression cycle, which is essentially time, while the *y*-axis represents the amplitude of signals with different magnitudes after normalization. Based on this, the hysteresis curve can be derived by recording the entire loading cycle, as shown in the figure. Compared with phase difference measurement, the displacement variables vary at different positions of the rubber, resulting in large errors in phase difference testing. Therefore, this article quantifies the energy dissipation by integrating the hysteresis curve. In addition, a coefficient correction is implemented to eliminate the inhomogeneity of energy dissipation at different positions, thus obtaining reliable dissipation data.

(4) $$W_{r}=\frac{\pi \cdot \sigma_{0}\cdot \varepsilon_{0}\cdot \sin\delta }{V_{r}}=\Delta \tau W_{ds}$$

In the formula, the parameter Δτ is the shape parameter, which is primarily intended to calibrate the differences in deformation between two different specimens caused by variations in volume and uniaxial deformation. Its calculation is mainly based on the volume and the deformation mode of the rubber material in the deformed regions of the two specimens.

Figure 2a presents the measured displacement–pressure data of the rubber isolator under tensile–compressive tests. Through normalization processing, the phase lag caused by the hysteresis behavior of the rubber material can be extracted. As shown in the figure, the phase lag corresponds to approximately 1/12 of a full cycle and is therefore calculated as $\frac{\pi }{6}$. Figure 2b illustrates the displacement–force relationship curves under different coordinate definitions. It can be observed that within a complete loading–unloading cycle, the displacement and force form a closed hysteresis loop. This figure provides a direct interpretation of the energy dissipation mechanism arising from the phase lag: by integrating the closed loop and calculating its enclosed area, the total energy dissipation per cycle under the given displacement condition can be obtained.

### 2.3. Quantification Method of Equivalent Damage Parameters

In the structure of rubber vibration dampers, the rubber material undergoes reciprocating compressive deformation. Due to the stress hysteresis phenomenon of rubber polymer materials, the rubber converts kinetic energy into thermal energy generated by friction between molecular chains [40]. Owing to the long molecular chains of rubber and the dense cross-linked structure of vulcanized rubber, its energy conversion capacity is significantly enhanced, thereby achieving the effect of attenuating abrupt forces transmitted from other components. It can be concluded therefrom that the energy absorption (conversion) function of rubber is the primary reason for its excellent vibration-damping performance. In this study, the test conditions are determined by taking the positions with the maximum energy absorption of the two (rubber isolator and rubber test columns) as the most vulnerable points of the structure, which are also designated as the damage areas of the test pieces.

As evident from the simulation and experimental results in Section 2, the main compressed part of the rubber vibration damper is the metal interlayer, which is consistent with the main energy absorption part of the rubber test columns selected in the experiment. The vertical force is the dominant compressive load, while the shear force serves as a secondary load. From the perspectives of maximum equivalent strain energy density and total dissipated energy, the two components can be functionally equivalent. Based on this, this article proposes to couple the rubber vibration damper and rubber test columns to realize their equivalence from the perspective of the most vulnerable parts. Thus, the damage parameters of the two can be listed as equal:

(5) $$\Delta W_{t}=\Delta W_{s}$$

Based on the elaboration and simplification of the above two sections, it can be derived that

(6) $$\Delta \sigma_{t\max}\varepsilon_{t\max}+\tau_{t}\frac{W_{dt}}{V_{t}}=\delta \Delta \sigma_{s\max}\varepsilon_{s\max}+\tau_{s}\frac{W_{ds}}{\pi r^{2}h}$$

where $\Delta \sigma_{\max}\varepsilon_{\max}$ represents the maximum von Mises equivalent stress and strain per unit volume; and τ represents the correction parameter, which is mainly used to correct the volume and shape effects in the energy loss per unit volume. This is because for different rubber structural components, the stress and strain vary at different positions during compression and tension, leading to different energy losses. According to the simulation results of the elastic isolator, its main deformation and stress concentration occur in the middle annular region. Therefore, it can be simplified by assuming consistent damage energy at each position of this region, and only the volume factor needs to be excluded. For the elastic test cylinder, the hourglass-shaped deformation requires sectional calculation, as the main deformation concentrates at the relatively thin central part. Thus, other regions are excluded to focus the calculation on this part and eliminate the volume effect. δ denotes the correction of the equivalent strain energy density, which is mainly reserved for parameter correction in future studies where ultra-high loads may lead to structural failure under low-cycle fatigue. $W_{d}$ stands for the total energy dissipation of the entire rubber part within a single cycle.

## 3. Results

**Figure 3:**
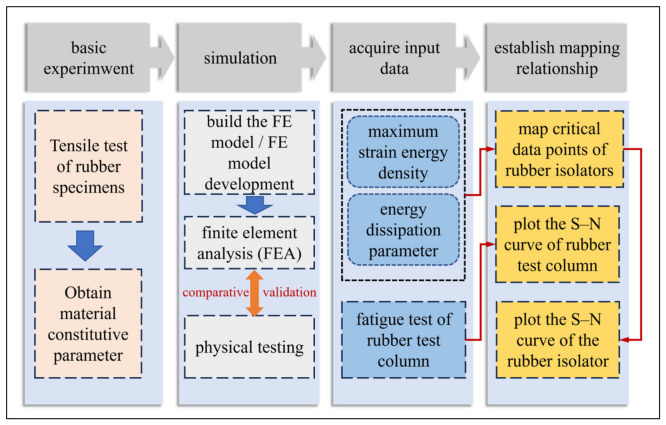
Flowchart for simulation and experiments.

The overall research procedure of this section is illustrated in Figure 3. In the fundamental experimental stage, the dumbbell-shaped rubber specimen is first taken as the research object, and its raw mechanical response data are obtained through uniaxial tensile tests. Considering the nonlinear mechanical characteristics of the rubber material, reasonable theoretical assumptions are proposed, relevant formulas are derived, and the key material parameters are determined, thereby establishing a constitutive equation suitable for this rubber material. To verify the reliability of the established constitutive model, mechanical property tests are conducted on both the rubber specimen column and the rubber isolation bearing. Meanwhile, the simulation boundary conditions and test environmental parameters are set according to their actual service conditions, and corresponding simulation analyses are performed. The validity of the model is confirmed by comparing the simulation results with the test data. On the basis of the above model verification, further systematic simulation tests are carried out under multiple designed loading conditions, while corresponding fundamental cyclic tests are also conducted to obtain critical data from both approaches. Subsequently, systematic fatigue tests are performed on the rubber specimen column, and its fatigue life curve is plotted based on the test data. By utilizing the mapping relationship established in the second part of this study, the fatigue performance of the specimen column is equivalently transformed to that of the rubber isolator. Consequently, the fatigue data points of the isolator are derived from those of the specimen column, and the fatigue life prediction curve for the rubber isolator is constructed accordingly.

### 3.1. Test Specimens and Equipment

**Figure 4:**
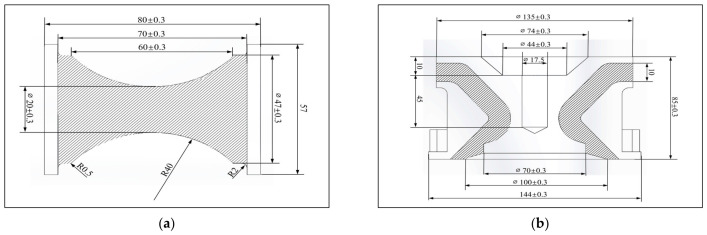
The dimension drawing of the rubber specimen column model (**a**), and the dimension drawing of the rubber isolator model (**b**).

The test columns adopted in this study were fabricated from a natural rubber material with the identical formulation and manufacturing process as that of the elastic vibration dampers. The rubber material formulation consists of natural rubber, a vulcanizing agent, an anti-aging agent, an antioxidant, an adhesive, a filler, and other additives. The two ends of the rubber were vulcanized to bond with the same aluminum alloy material as that of the vibration dampers, which effectively reproduces and achieves the structural equivalence of the original component. The ANSYS simulation software was employed for numerical simulations, while the MTS testing machine was used for mechanical tests with standard clamps and a self-designed special fixture for elastic vibration dampers. For fatigue tests, a modified MTS testing machine was utilized; to reduce test costs, a three-station fixture was designed for rubber test columns and a single-station fixture for vibration dampers, respectively. The test cylinder employed in this study is made of natural rubber, with the same formulation and manufacturing process as the elastic isolator. The rubber compound consists of natural rubber, a vulcanizing agent, an anti-aging agent, an antioxidant, an adhesive, a filler, and other additives, among which the anti-aging agent and adhesive are specially formulated composite reagents. The rubber isolator is mainly composed of two materials: aluminum alloy and natural rubber. Its upper part is a square with a side length of 144 mm, with through holes at the four corners of the top surface, mainly used for engine mounting. The lower part is cylindrical with internal threads for connecting to the vehicle chassis. The aluminum alloy serves to encapsulate the elastic material and connect to the vehicle structure, while the natural rubber presents an annular hourglass shape embedded with the aluminum alloy, mainly undertaking the connection between the upper and lower aluminum parts and the vibration isolation function. The overall dimension is 144 mm × 144 mm × 85 mm. The rubber test cylinder consists of two main parts, with its geometry designed in accordance with ASTM standards. Both ends are aluminum alloy support plates of 57 mm × 57 mm × 5 mm, and the middle part is a 70 mm high hourglass-shaped rubber section. The two parts are bonded through rubber vulcanization, using the same process as the rubber isolator. The detailed geometry is shown in Figure 4 in the simulation section. Simulations were performed using ANSYS 2023 R1, while mechanical tests were carried out on an MTS testing machine equipped with standard fixtures and a self-designed special fixture for the elastic isolator.

**Figure 5:**
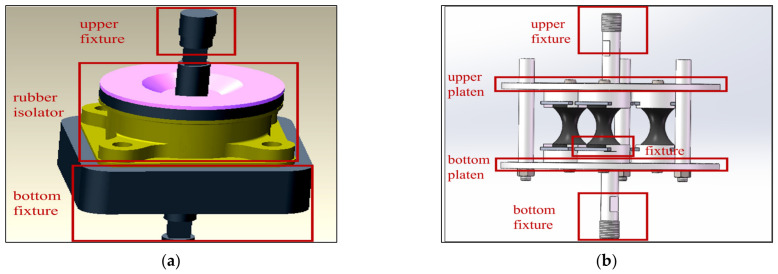
Special fixture for rubber isolator (**a**) and rubber test cylinder (**b**).

Figure 4 presents the specific dimensional data of the two objects studied in this work. The rubber parts of both are based on circular geometries: the rubber specimen column is mainly dumbbell-shaped and manufactured in accordance with ASTM standards, while the rubber part of the isolator is mainly hourglass-shaped, as indicated by the shaded areas in the figure. A modified MTS testing machine was used for fatigue tests. To reduce experimental costs, a three-station fixture for rubber test cylinders and a single-station fixture for isolators were designed separately. The designs of both fixtures are presented in Figure 5.

### 3.2. Establishment of the Simulation Model

Owing to the fact that the stiffness of the metal outer shell and inner liner is much higher than that of the internal rubber and that the research object is a rubber material, the external structure can be simplified. The Mooney–Rivlin hyperelastic constitutive model is adopted to construct the rubber material model. In the Mooney–Rivlin model, considering the incompressibility of rubber materials, the strain energy function of hyperelastic materials can be expressed as [39]:

(7) $$W_{s}=W_{d}(I_{1},I_{2},I_{3})+W_{b}(J)=\sum_{i+j=1}^{N}C_{ij}{\left(I_{1}-3\right)}^{i}{\left(I_{2}-3\right)}^{j}+\sum_{k=1}^{N}\frac{1}{d_{k}}{\left(J-1\right)}^{2k}$$

where $I_{1},I_{2},I_{3}$ denote the first, second, and third strain tensor invariants, respectively; $W_{d}(I_{1},I_{2},I_{3})$ represents the function of strain tensor invariants $I_{1},I_{2},I_{3}$; $W_{b}(J)$ is the function of volume ratio J; N stands for the expansion order of the constitutive model expression; $C_{ij}$ denotes the material constant (i, j take the value of 0 or 1); and $d_{k}$ is the incompressibility parameter of the material (k takes the value of 0 or 1). Considering the incompressibility of the material, the strain potential energy corresponding to $I_{3}$ has no effect, and only the first and second strain tensor invariants are adopted when defining the deviatoric term $W_{d}$.

Incompressibility of rubber: the volumetric deformation is zero, satisfying the condition that $\varepsilon_{11}+\varepsilon_{22}+\varepsilon_{33}=0$.

The constitutive relation of hyperelastic materials can be described by the strain energy density function, and the above equation can be simplified to derive the strain energy density function model of the Mooney–Rivlin model, with the typical two-term expansion expressed as:

(8) $$W=C_{10}(I_{3}-3)+C_{01}(I_{2}-3)+\frac{1}{d}{\left(J-1\right)}^{2}$$

Based on the differential relationship between strain energy density and stress, the relationship between stress and elongation ratio can be defined as:

(9) $$\sigma =2(1-\frac{1}{\lambda^{3}})(C_{10}\lambda +C_{01})$$

Through transposition and simplification, the expression for the mechanical properties of hyperelastic materials based on the two-parameter Mooney–Rivlin constitutive model can be derived as follows:

(10) $$\frac{\sigma }{2(\lambda -\frac{1}{\lambda^{2}})}=\frac{1}{\lambda }C_{01}+C_{10}$$

**Figure 6:**
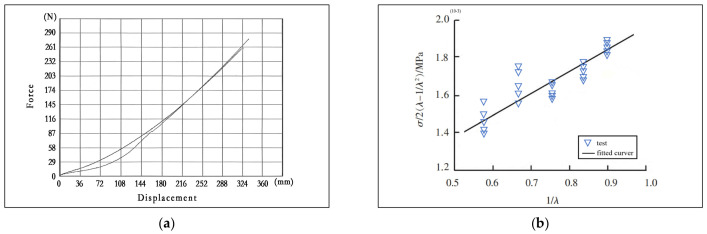
Tensile data of dumbbell rubber specimens (**a**), and the two-parameter fitting curve (**b**).

Uniaxial tensile tests were conducted on rubber specimens at room temperature (22 °C), and the experimental data shown in Figure 6a were obtained. To ensure that the measured data truly reflect the stable constitutive parameters of the material, pre-tensile tests were carried out before the formal experiments, following the same loading procedure as the formal tests; the results of the pre-tests are also presented in the figure. A total of five replicate tests were performed. According to Equation (10), the relevant values were taken, and the corresponding calculations were carried out. At each stretching ratio λ, five data points were selected, and the calculated results were plotted in Figure 6b. A linear fit was then applied to these data points. The slope and intercept of the fitted line correspond to the two required material parameters, namely C_10_ and C_01_ in the Mooney–Rivlin model.

Substituting the stress values measured σ at different elongation ratios λ in the experiment into the above equation yields discrete data points, from which the material parameters can be obtained via curve fitting: *C*_10_ = 0.1694, *C*_01_ = 0.0023. The simulation temperature was set at 22 °C, with the incompressibility parameter taken as *D*_1_ = 0.0001 MPa [41].

**Figure 7:**
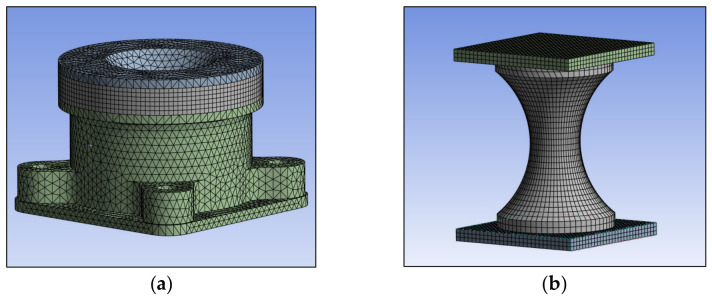
Mesh of the rubber isolator (**a**) and the rubber test cylinder (**b**).

In the material setup of the established model, consistent with the actual elastic support, the rigid structures at both ends are assigned aluminum alloy, while the hyperelastic part is natural rubber, adopting the Mooney–Rivlin 2-parameter model as the basic constitutive model. The contact condition is set to no slip. Different types of meshes are generated according to structural characteristics and material properties. To ensure simulation accuracy, the rubber region is refined, and irrelevant features are simplified. The meshing results are shown in Figure 7. Boundary and loading conditions are applied in accordance with actual service conditions: the rubber test cylinder is constrained via the upper and lower support plates, and the rubber isolator is fixed through the upper through-holes and lower threaded holes, with tests conducted under actual working conditions.

In the simulation, tetrahedral meshing is mainly used for the metal parts. This is because the deformation and failure rate of the metal parts are the lowest both in simulation and actual service. Hence, tetrahedral elements are adopted for the metal parts. For the rubber parts, due to their large deformation, hexahedral meshing is employed to ensure test accuracy. To verify mesh sensitivity, the following study was conducted: simulation results for the metal parts show that the mesh size has almost no effect on the test results; the mesh sensitivity of the rubber parts is analyzed and validated herein.

The simulation equipment used is a Lenovo Legion Y7000 laptop, with ANSYS 2023 R1 software. The detailed test results are shown in Table 1 below.

**Figure 8:**
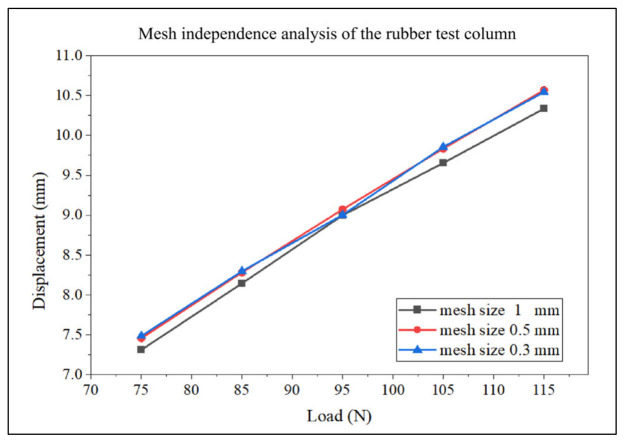
Mesh independence analysis of the rubber test column.

**Figure 9:**
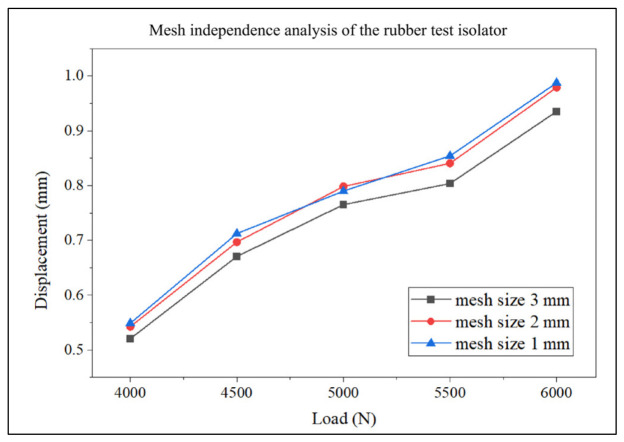
Mesh independence analysis of the rubber isolator.

Regarding the mesh independence analysis, this study first conducted simulation tests under severe loading conditions, i.e., under fixed loading parameters; different mesh sizes were generated following standard practices, and simulations were performed to observe the most fundamental displacement data. Since displacement is the basic parameter for calculating other quantities and can be directly compared with experimental measurements to reflect the accuracy of the simulation model, it was adopted as the primary indicator for mesh convergence assessment. As shown in Table 1, a mesh size of 0.5 mm was used for the rubber part of the specimen column, while a mesh size of 2 mm was adopted for the rubber isolator. To further verify the independence of the results from mesh size under various loading conditions, additional simulations were carried out with several mesh sizes that yielded similar outcomes, and the results are presented in Figure 8 and Figure 9. The calculated results show that the maximum relative error for the rubber specimen column is 2.2%, with the corresponding maximum average relative error of the curve being 1.7%; for the rubber isolator, the maximum relative error is 4.5%, and the maximum average relative error of the curve is 4.1%.

From the test results, it can be concluded that for the rubber isolator, a mesh size of 4 mm for the metal parts and a primary mesh size of 2 mm for the rubber parts are appropriate. For the rubber specimen column section, the metal parts adopt a mesh size of 0.4 mm (still tetrahedral), while the rubber parts adopt a mesh size of 0.5 mm (hexahedral). This effectively demonstrates that the test results are essentially independent of the meshing strategy. Therefore, the above meshing strategy is adopted.

**Figure 10:**
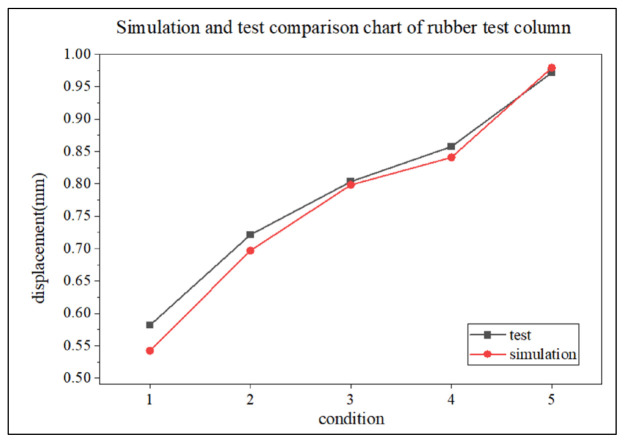
Comparison of simulation and experimental results for the rubber test column.

**Figure 11:**
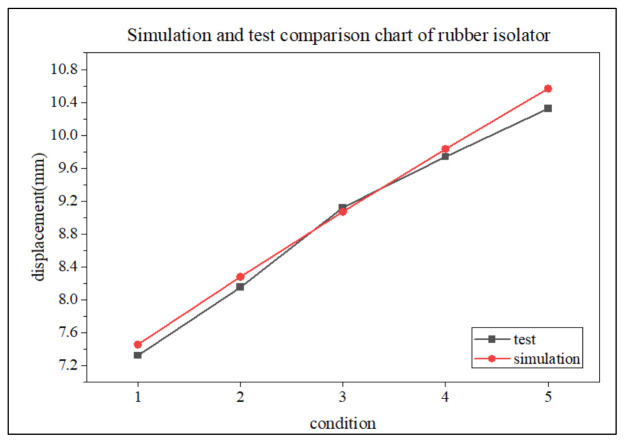
Comparison of simulation and experimental results for the rubber isolator.

A comparison between the static simulation results and the actual test data is presented in Table 2. It is evident from the data that the Mooney–Rivlin (MR) model established by this method exhibits a good fitting performance for the structural components in this experiment. By comparison between simulation and experimental results, it is concluded that the error of the constitutive model is only 2–7%. Meanwhile, the plotted curves indicate that the proposed model can well reflect the stress–strain evolution process of rubber materials under loading (Figure 10 and Figure 11).

**Figure 12:**
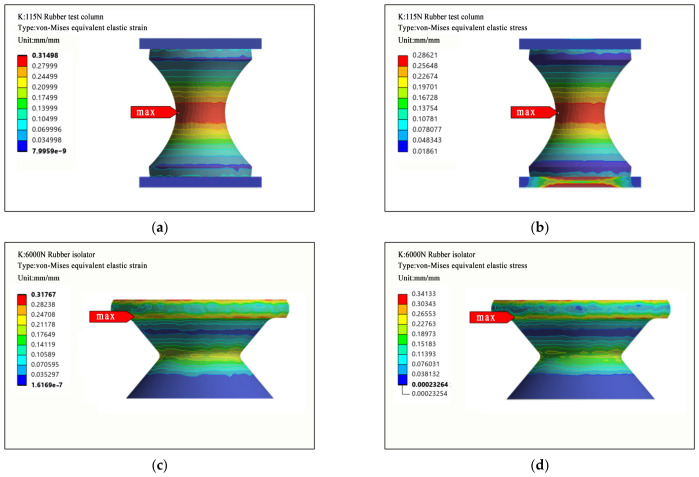
Equivalent strain simulation result of the rubber test column (**a**); equivalent stress simulation result of the rubber part of the rubber test column (**b**); equivalent strain simulation result of the rubber part of the rubber isolator (**c**); and equivalent stress simulation result of the rubber part of the rubber isolator (**d**).

For the convenience of description and experimental implementation, the test conditions for the two cases were each divided into five levels, and were designated as A1 to A5 and B1 to B5, respectively, in ascending order of loading from the mildest to the most severe. Figure 12 presents the von Mises equivalent stress–strain curves obtained from the simulation analysis for the two types of test specimens (Table 3).

Table 4 presents the measured values of dissipated energy for the research object and its equivalent test pieces in this study.

**Figure 13:**
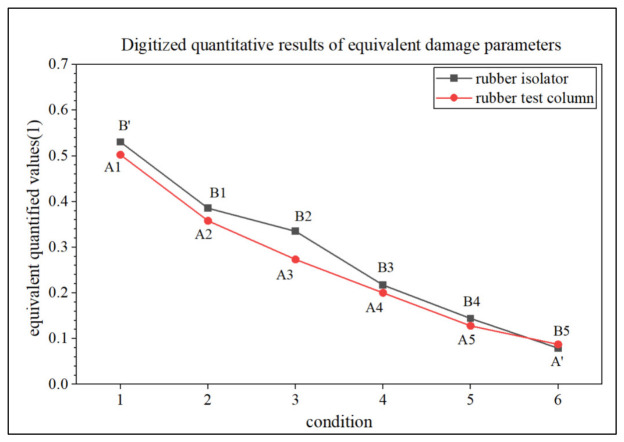
Quantification of equivalent damage parameters for rubber test specimens and rubber isolators.

Based on the aforementioned simulation and experimental results, calculations were performed according to the method established in Chapter 2 to obtain the maximum strain energy density and the energy dissipation per unit volume. Further analysis and computation were then carried out using Equation (6), resulting in Table 5. Subsequently, the two sets of results were plotted as curves and adjusted in accordance with the sequence of test working conditions, yielding the curves presented in Figure 13. It should be noted that A’ and B’ in the figure represent the extrapolation extensions of the original curves following their respective trends. This extrapolation treatment is intended to align the equivalent numerical quantities of the two different specimens under various working conditions, thereby facilitating the application of the established mapping relationship.

### 3.3. Rubber Test Cylinder Fatigue Test

**Figure 14:**
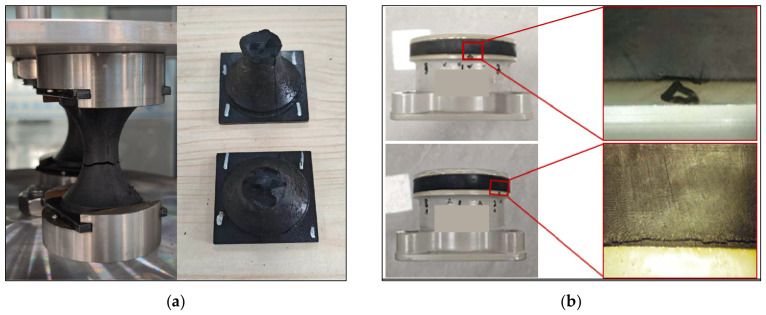
Test result diagram of the rubber test column (**a**), and the test result diagram of the rubber isolator (**b**).

Figure 14 presents the experimental results. By comparing with the locations of the maximum equivalent stress obtained from the simulation in Figure 12, it can be seen that the damaged regions in the tests all correspond to the positions where the equivalent strain energy density reaches its maximum in the simulation, which further validates the reliability of the simulation.

Fatigue life tests on rubber specimens were conducted using a modified MTS testing machine. The fixtures were installed sequentially from bottom to top, and different displacement conditions were defined according to the actual service conditions. It should be noted that, to match real-world application scenarios, a strain ratio of R < 0 (i.e., compression-dominated loading) was adopted. To minimize the Mullins effect, displacement control was employed, and the test frequency was set to 3–4 Hz, in order to reduce the potential influence of rubber aging caused by temperature rise on the test results. To balance test efficiency and cost control, six specimens were tested under each displacement condition, totaling 30 fatigue tests completed. The test termination criterion was the failure of the rubber elastomer, which was defined as the appearance of obvious cracks on the surface or a significant drop in stress during the reciprocating cycle.

The test results are presented in Table 6, showing the fatigue test data of the rubber test cylinder. The results indicate that the fatigue life of this rubber material exhibits a certain degree of dispersion. Specifically, under condition C5, no obvious cracks appeared on the specimens after 1.2 to 1.5 million reciprocating tensile cycles. According to industry-wide standards, the service life of the rubber isolator under such working conditions is classified as infinite.

**Figure 15:**
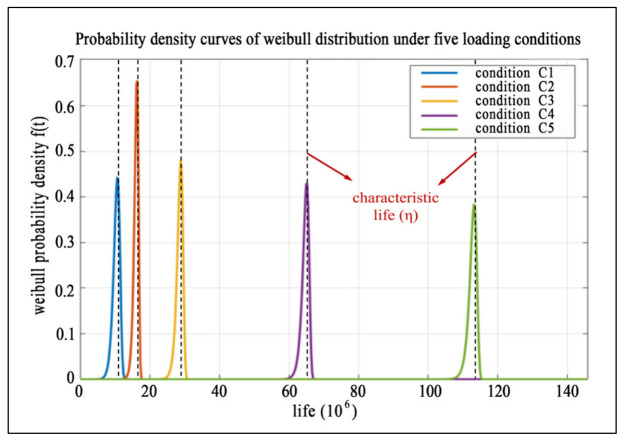
Weibull probability density curves.

Through the analysis of fatigue and mechanical performance of rubber specimen columns under various working conditions, the fatigue test data exhibit considerable scatter. Since the fatigue life of rubber materials generally conforms to the Weibull distribution, the measured data were fitted using the Weibull distribution. The specific procedure is as follows: the test data are first sorted in ascending order, and the empirical failure probabilities are estimated using the median rank method. Subsequently, linearization is performed based on the two-parameter Weibull cumulative distribution function, followed by linear regression fitting using the least squares method. The shape parameter and scale parameter are then back-calculated from the slope and intercept of the fitted straight line. The calculation results indicate that the shape parameters under all working conditions are greater than 1, suggesting a wear-out failure mode. Furthermore, as the working conditions become progressively milder, the shape parameter tends to increase, implying that under more severe working conditions, the rubber material is more prone to random failures. The calculation results are presented in Figure 15, with detailed parameters summarized in Table 7.

**Figure 16:**
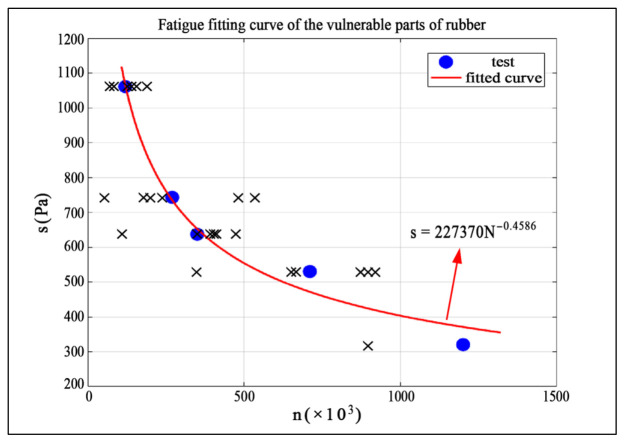
Fatigue life curve of the rubber test column (S–N curve).

The fatigue life curve of the rubber test columns measured in this study is primarily based on the Wöhler equation $S=a\cdot N^{-b}$ as the basis function. From this, five sets of valid data of the rubber test columns were obtained for fatigue life curve fitting, which was mainly conducted by plotting a double logarithmic curve, and the S-N curve was finally derived. The goodness of fit $R^{2}$ reached 0.9622, and the final fitting curve equation is presented as $S=227370N^{-0.4586}$ (Figure 16).

## 4. Discussion

**Figure 17:**
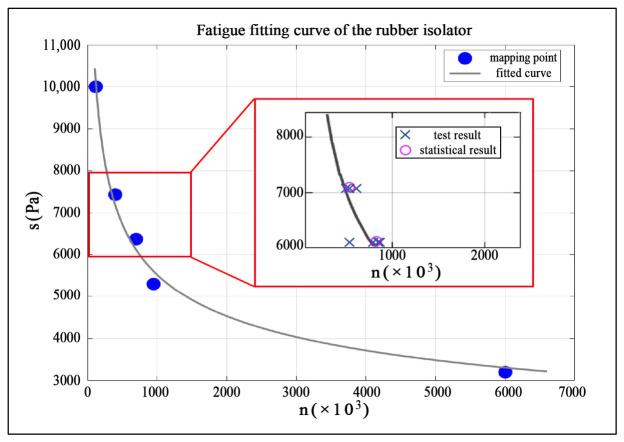
Equivalent fatigue life curve of rubber damper (S–N curve).

Based on the equivalence relationship in the second part, the S-N curve of the rubber test column was subjected to the corresponding equivalence processing. The measured data points from the tests were adopted, with the special case of working condition C5 excluded. A reasonable extrapolation was conducted to supplement the data of working condition C5 in accordance with the general properties of rubber materials. The data was fitted using the double logarithmic fitting method. Based on this, the fatigue life curve was fitted, and Figure 17 was drawn. The goodness of fit, $R^{2}$, was 0.9728, and the fitted curve equation was $S=287430N^{-0.2861}$.

To observe the curves, the working conditions D1 and D2 were applied to the rubber isolator, where the corresponding stress amplitudes were set to 6045 and 7024, respectively. The fatigue lives of the rubber isolator under different working conditions obtained from the curves are presented in Table 8. For this test, three valid fatigue life tests of the rubber isolator were conducted under the same working conditions. The experiments were carried out on an MTS testing machine with a position control strategy. Due to the small relative displacement of the rubber isolator, the test frequency was set to 6 Hz to reduce experimental costs. A total of six valid experiments were performed in this verification test.

The experimental results of this study are summarized in Table 9. Owing to the discrete life characteristics of rubber materials, the third set of data under working condition D1 exhibited significant dispersion. To avoid compromising the accuracy of the test results, given the limited number of experimental groups, a fourth test was conducted. After removing the bad data, the actual fatigue lives under the two working conditions were calculated using statistical methods.

It can be clearly seen from the test results that the method of transferring the fatigue life curve of the rubber test cylinder to the rubber isolator of the same material using the two-parameter mapping relationship of “maximum strain energy density + cyclic energy dissipation” exhibits favorable mapping accuracy and reliability.

This result not only verifies the effectiveness of the proposed mapping method in predicting the fatigue life of complex rubber structural components but also further confirms that the maximum strain energy density and cyclic energy dissipation are the core key factors characterizing the fatigue life of rubber materials, providing solid experimental support for the fatigue performance evaluation of rubber structural components.

## 5. Conclusions

Through systematic theoretical derivation, numerical simulation, and experimental validation, the following main conclusions are obtained: the maximum strain energy density and cyclic dissipated energy in uniaxial tests are identified as key indicators for characterizing the fatigue life of rubber, providing a theoretical basis for fatigue evaluation of rubber structural components; the Mooney–Rivlin constitutive model established from uniaxial tensile tests exhibits good agreement between simulation and measured data, with a maximum error below 7% and an average error less than 3%, and can accurately locate vulnerable regions to guide structural optimization; by means of the established mapping relationship, the fatigue life curve of rubber specimens is successfully transferred to isolators made of the same material, with prediction errors not exceeding 8%, enabling effective prediction of weak locations and fatigue life. Furthermore, the combined “simulation + experiment” approach adopted in this study allows the life curve of complex structural components to be derived solely from standard specimen fatigue data, significantly reducing testing costs and development cycles, thus providing a practical and valuable technical pathway for the design optimization, performance prediction, and R&D process improvement of similar rubber components, with considerable engineering application value.

Meanwhile, there remain several aspects of this study that can be further improved. The current mapping model is only applicable to rubber components made of the same material and with similar structures, and special loading conditions, such as torsion, have not been considered under actual operating conditions, so the applicability of the model needs to be further expanded. Key aging factors in actual service environments, such as salt spray corrosion and temperature aging, have not been considered, so the alignment with real-world conditions needs further improvement [42]. Boundary conditions in the simulation analysis have been simplified, and complex loading conditions such as impact loads have not been taken into account. Based on the above limitations, future research can be pursued in the following directions: expand the applicability of the mapping model to enable fatigue performance transfer across different materials and structural forms of rubber components; incorporate aging factors such as salt spray and temperature to establish a fatigue life prediction model that better matches actual service environments; and refine boundary condition settings to include complex operating conditions, such as impact and vibration coupling, thereby further enhancing the engineering applicability and prediction accuracy of the model.

## Figures and Tables

**Table 1:** Mesh independence analysis results.

Rubber Test Column					Rubber Isolator				
Load/N	Mesh Size/mm	Test Result (Displacement/mm)	Relative Change	Solution Time/h	Load/N	Mesh Size/mm	Test Result (Displacement/mm)	Relative Change	Solution Time/h
115	3	7.4214	-	0.05	6000	5	-	-	-
115	2	9.8457	32.7%	0.18	6000	4	0.8534	-	0.8
115	1	10.3342	4.9%	0.3	6000	3	0.9352	9.6%	1.5
115	0.5	10.5672	2.2%	0.5	6000	2	0.9791	4.6%	2.5
115	0.3	10.5427	0.3%	0.8	6000	1	0.9875	0.9%	6

**Table 2:** Simulation results of the rubber damper and the rubber test column.

Rubber Isolator			Rubber Test Column		
Load/N	Test (Displacement/mm)	Simulation (Displacement/mm)	Load/N	Test (Displacement/mm)	Simulation (Displacement/mm)
4000	0.5818	0.5425	75	7.325	7.4588
4500	0.7213	0.6968	85	8.154	8.2817
5000	0.8034	0.7983	95	9.124	9.0727
5500	0.8574	0.8407	105	9.739	9.8339
6000	0.9714	0.9791	115	10.324	10.567

**Table 3:** Simulation results of rubber test columns and rubber vibration dampers.

Rubber Test Column			Rubber Isolator		
Load/N	Maximum Von Mises Equivalent Elastic Strain	Maximum Von Mises Equivalent Stress/MPa	Load/N	Maximum Von Mises Equivalent Elastic Strain	Maximum Von Mises Equivalent Stress/MPa
75	0.31498	0.28621	6000	0.31767	0.34133
85	0.29178	0.26659	5500	0.29250	0.31338
95	0.26784	0.24625	5000	0.26957	0.2852
105	0.24316	0.20313	4500	0.24462	0.25705
115	0.21773	0.22512	4000	0.21907	0.22878

**Table 4:** Integral results of single-cycle hysteresis curves for rubber test columns and dampers.

Rubber Test Column Conditions (Load/N)	Energy Loss/J	Rubber Isolator Conditions (Load/N)	Energy Loss/J
115	2.64710	6000	5.41825
105	1.94268	5500	2.93733
95	1.41062	5000	2.58135
85	0.69244	4500	1.54471
75	0.37419	4000	0.87098

**Table 5:** Quantitative results of equivalent damage parameters.

Rubber Test Column				Rubber Isolator			
Condition (Load/N)	Equivalent Strain Energy Density	Energy Loss per Unit Volume per Cycle	Equivalent Quantified Values	Condition (Load/N)	Equivalent Strain Energy Density	Energy Loss per Unit Volume per Cycle	Equivalent Quantified Values
115 (A1)	0.09015	0.39421	0.50239	6000 (B1)	0.09166	0.29373	0.38539
105 (A2)	0.07779	0.26471	0.35805	5500 (B2)	0.07688	0.25814	0.33502
95 (A3)	0.06596	0.19427	0.27341	5000 (B3)	0.06288	0.15447	0.21735
85 (A4)	0.04939	0.14106	0.20033	4500 (B4)	0.05711	0.08710	0.14422
75 (A5)	0.04902	0.06924	0.12806	4000 (B5)	0.03608	0.04327	0.07935
A’	-	-	0.08243	B’	-	-	0.51872

**Table 6:** Fatigue test results of the rubber test column under five working conditions.

Condition/mm	Test Cycle					
	1	2	3	4	5	6
C1	74,532	132,384	132,495	163,452	123,532	83,479
C2	21,426	174,867	193,457	486,542	542,003	235,431
C3	63,248	394,213	394,952	468,342	348,624	395,247
C4	800,103	650,013	334,867	854,312	684,527	897,534
C5	843,251	-	-	-	-	-

**Table 7:** Weibull distribution results of rubber test cylinder fatigue tests.

Condition	Shape Parameter (m)	Characteristic Life (η)	Core Life Span
C1	1.28	106,160	118,300
C2	2.95	160,100	275,600
C3	3.75	285,540	344,100
C4	7.76	647,830	703,500
C5	-	-	1,200,000

**Table 8:** Fatigue test results of the rubber damper under the most severe working conditions.

Condition/mm	Test Cycle			
	1	2	3	4
D1	800,000	752,047	553,482	798,524
D2	448,273	483,251	458,761	—

**Table 9:** Comparison results of verification tests.

Condition	Stress Amplitude	Fitting Mapping Lifetime	Actual Service Lifetime	Prediction Error
D1	6045	725,000	783,523	7.47%
D2	7030	432,000	463,428	6.93%

## Data Availability

The data presented in this study are available on request from the corresponding author. The data are not publicly available due to privacy and confidentiality agreements with the industrial partners.
